# Revealing Structure–Property Coupling During Thermal Conversion of Metal–Organic Frameworks

**DOI:** 10.1002/smll.74631

**Published:** 2026-07-22

**Authors:** Gourav Bhattacharya, Sameh Khalil, Wajahat Khalid, Indrianita Lionadi, Abhijit Ganguly, Anna L. Eichhorn, Christian Dietz, Amir Farokh Payam, Supriya Chakrabarti

**Affiliations:** ^1^ Nanotechnology and Integrated Bioengineering Centre School of Engineering Ulster University Belfast UK; ^2^ Physics of Surfaces Institute of Materials Science Technische Universität Darmstadt Darmstadt Germany

**Keywords:** atomic force microscopy, calcination, electronic structure, energy transformation, engineering, materials science, nanocomposite, nanoporous, nanoscopic scale, nanotechnology

## Abstract

Metal–organic frameworks (MOFs) provide powerful templates for constructing porous inorganic materials, yet the nanoscale thermal conversion pathways remain unclear. We reveal the mechanistic origins of structure–property emergence during thermal transformation of Cu‐BTC (HKUST‐1 MOF) into nanoporous copper oxides, uncovering transient nanocomposite states that dictate the mechanical and optoelectronic properties. By integrating various characterization techniques, we establish a unified multiscale framework linking phase evolution, pore architecture, nanomechanics, and electronic structure. Controlled calcination (300°C–500°C) transforms MOF to nanoporous oxide with non‐monotonic mechanical evolution. At 300°C, partial decomposition produces a heterogeneous Cu_2_O/CuO‐carbon nanocomposite that retains mesostructural connectivity and exhibits high local Young's modulus (∼41 GPa). This reinforcement arises from residual carbon networks, heterophase interfaces, and mesoporosity. At 500°C, phase‐pure porous CuO forms with improved optical absorption but reduced stiffness due to pore coarsening and sintering. These results reveal that functional performance in MOF‐derived oxides is not simply governed by bulk phase composition but by transient nanoscale connectivity and heterogeneity formed during partial conversion. This establishes a structure–property–function paradigm for MOF‐templated oxides, demonstrating that controlled intermediate states provide a powerful route for engineering mechanically robust and optoelectronically active nanoporous semiconductors for catalysis, sensing, and energy conversion applications.

## Graphical abstract

Multifrequency atomic force microscopy (AFM) combined with hybrid density functional theory (DFT) and complementary spectroscopic techniques uncovers transient nanoscale states during Cu‐BTC MOF conversion. Intermediate Cu_2_O/CuO‐carbon nanocomposites maximize mechanical robustness and functionality, while fully oxidized porous CuO enhances light absorption, which confirms a structure–property–function framework for advanced MOF‐derived porous oxide engineering.

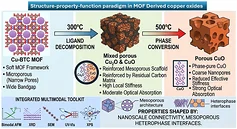

## 1 Introduction

Advancing materials with enhanced functionality is critical for enabling next‐generation technologies. Metal‐organic frameworks (MOFs) and their derivatives are a promising new class of materials with advanced functional characteristics beneficial for a wide range of applications, from energy and catalysis to biomedical and health care technology [1, 2, 3, 4]. MOFs offer a natural advantage for both mechanism studies and performance optimization [5, 6, 7, 8]. These new types of porous crystalline materials possess ultrahigh surface area and offer easy tuneability of physicochemical properties, and can be transformed into various derivatives by a simple calcination process. Now the optimization of the MOF‐derived structures requires a detailed understanding of the transformation mechanism in terms of crystalline phase evolution, morphology, electronic, and nanomechanical properties, which is still poorly understood and requires significant exploratory attention. The detailed understanding of the transformation mechanism of MOF to its derivatives unfolds the possibility of further optimization of the derived structures suitable for desired applications. Within this diverse family, copper‐based MOFs such as Cu‐BTC (HKUST‐1) have garnered particular attention as sacrificial templates for fabricating functional metal oxides, owing to their well‐defined porosity, morphological uniformity, and compositional precision [9, 10].

Recent advances have employed MOFs as precursors for synthesizing porous metal oxides (P‐MO*_x_*) [11, 12, 13], enabling precise control over structural and chemical features that are challenging to achieve via conventional synthesis [14]. This MOF‐derived approach capitalizes on the inherent order and tunability of frameworks like Cu‐BTC to produce P‐MO*_x_* with tailored pore architectures, hierarchical organization, and enhanced surface functionality [15].

Among these strategies, thermal decomposition stands out as a simple yet powerful method to convert MOFs into P‐MO*_x_* while retaining critical morphological features [16, 17]. However, the physico‐mechanical dynamics governing this transformation remain poorly understood [5, 18]. Parameters such as calcination temperature and heating rate (HR) exert a profound influence on phase stability, porosity, and mechanical behavior, yet their mechanistic impact has not been fully resolved [15]. A deeper understanding of these processes is essential for guiding the rational design of oxide nanomaterials with tunable functionalities.

In this study, we investigate the calcination‐induced transformation of Cu‐BTC into nanoporous copper oxides (P‐CuO*_x_*), elucidating how thermal parameters modulate crystalline phase evolution, morphology, electronic, and nanomechanical properties. We reveal a temperature‐driven transition from Cu_2_O‐rich mixed phases to P‐CuO within the 300°C–500°C range, accompanied by marked variations in porosity, surface stiffness, and energy dissipation behavior. These changes are captured using a suite of high‐resolution characterization techniques, including bimodal atomic force microscopy (AM‐AM and AM‐FM modes), scanning electron microscopy (SEM), X‐ray diffraction (XRD), X‐ray photoelectron spectroscopy (XPS), and ultraviolet–visible–near infrared (UV–Vis–NIR) spectroscopy. Furthermore, we have performed density functional theory (DFT) analysis to reveal and track the variations in electronic and mechanical properties of the transformation of Cu‐BTC into nanoporous copper oxides. The integration of our experimental approach with DFT simulation provides a comprehensive mechanistic model to explain MOF calcination with phase control and mechanical insights.

To move beyond qualitative analysis, we have incorporated advanced multivariate data approaches, specifically principal component analysis (PCA) and cross‐correlation mapping, to unravel the complex interdependence between structural and nanomechanical features. This data‐driven framework enables the extraction of quantitative relationships and reveals key insights into the coupled phase‐mechanical transformation mechanisms during calcination.

Our findings underscore the critical role of thermal conditions in governing the structural, mechanical, and functional evolution of MOF‐derived P‐CuO*_x_* materials. Notably, samples calcined under optimized conditions exhibit enhanced optical absorption across the visible spectrum, indicating their potential for photocatalysis and solar energy conversion. These samples also display distinct nanomechanical signatures, including increased surface stiffness and altered dissipation behavior. These correlated changes in optical, electronic, and mechanical properties reflect the underlying modifications in phase composition and pore architecture, which reinforce the importance of controlled thermal treatment in engineering multifunctional nanomaterials.

## 2 Methodology

**FIGURE 1:**
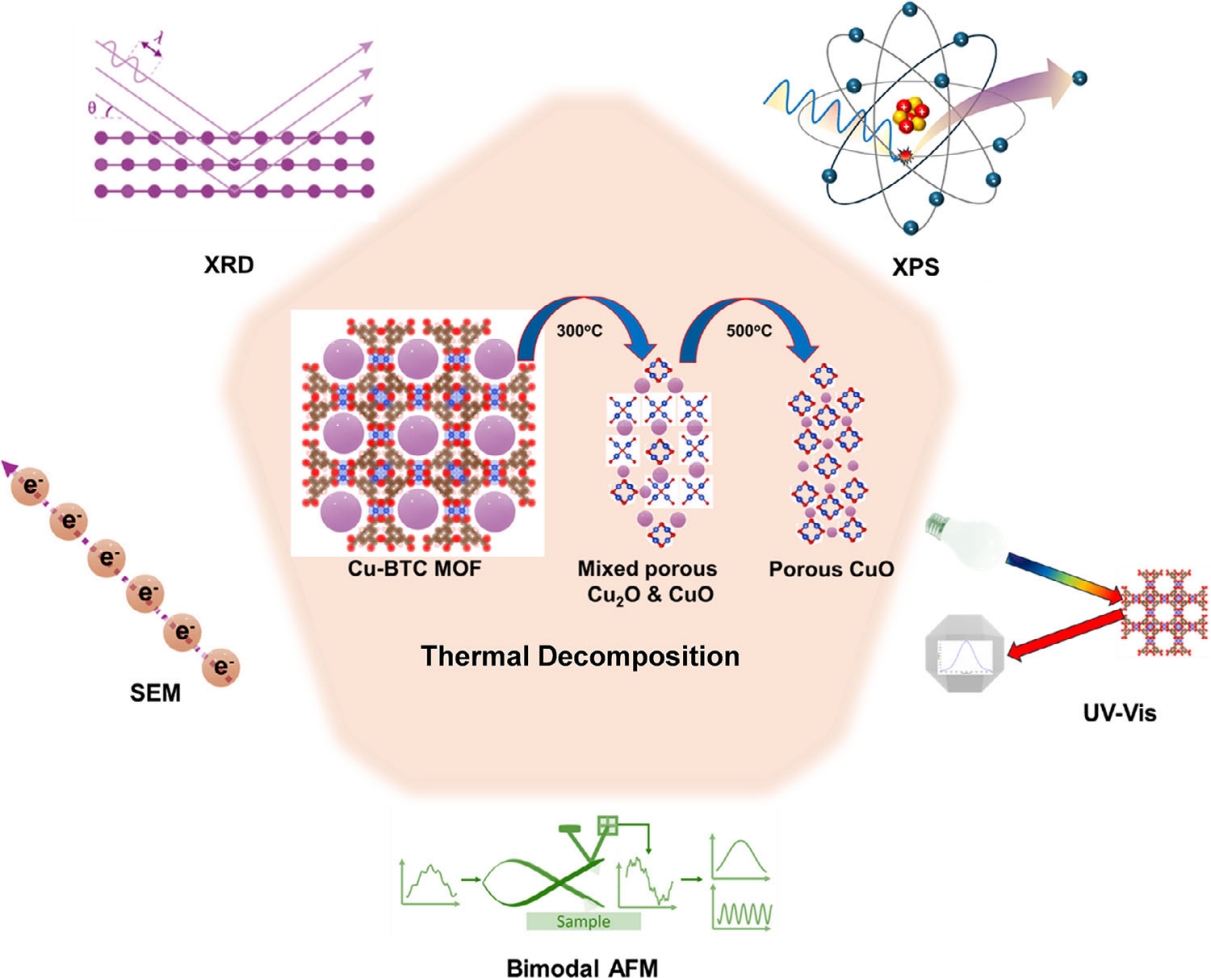
Schematic of the thermal evolution of Cu‐BTC metal–organic frameworks into porous nanoparticles and the corresponding multimodal characterization strategy. The illustration outlines the controlled thermal transformation of crystalline Cu‐BTC into porous nanoparticles, accompanied by changes in morphology, structure, and electronic properties. Morphological and nanoscale properties are investigated using scanning electron microscopy (SEM) and bimodal atomic force microscopy (AFM), while chemical composition and bonding are analyzed by XPS. Structural evolution is assessed by XRD, and optical responses are probed using UV–Vis–NIR spectroscopy. Experimental results are complemented by DFT simulations and PCA to provide atomistic insight and identify key correlations across the multimodal dataset.

To engineer porous copper oxides (P‐CuO*_x_*) with controlled structural and mechanical characteristics, we employed Cu‐BTC as a precursor and subjected it to a carefully modulated thermal decomposition process within a muffle furnace. The transformation was conducted in an air atmosphere, with critical calcination parameters such as calcination temperature (*T_n_*), HR, and dwell time to unravel their impact on phase evolution and material performance. A schematic overview of the experimental workflow is presented in Figure 1, which delineates each stage of synthesis and characterization. This includes advanced imaging and spectroscopic techniques such as SEM, XRD, XPS, UV–Vis–NIR spectroscopy, and crucially, the bimodal atomic force microscopy (AFM) for nanomechanical profiling. The schematic underscores the integrative strategy used to map both morphological and mechanical transformations throughout the calcination process.

Calcination was carried out at two representative temperatures, 300°C and 500°C, which were strategically chosen [15] to probe the temperature dependence of the Cu‐BTC to P‐CuO*_x_* transformation. These temperatures were selected based on the TGA/DTG profile of Cu‐BTC (Figure S1a), which shows the main framework decomposition occurring between approximately 285°C and 335°C, with a total mass loss of ∼66.4%. Therefore, 300°C was chosen to capture the intermediate transformation stage, while 500°C was selected to represent the fully converted oxide state after completion of the main decomposition process. Quantitative assessment of transformation efficiency was achieved by measuring sample mass before and after calcination (Figure S1b), thereby tracking weight loss as a proxy for decomposition extent and porosity development [15]. The selected temperatures were guided by the TGA/DTG profile and the temperature‐dependent transformation pathway as previously reported [15]. Subsequent structural and compositional analyses were performed using SEM, XRD, and XPS, providing insights into crystallinity, phase purity, and oxidation states. The optical characteristics were analyzed using a UV–Vis–NIR spectrometer to assess the light absorption capacity, which is crucial for various applications, including photocatalysis and solar energy conversion. To probe the nanomechanical evolution, bimodal AFM (AM‐AM and AM‐FM modes) was employed, enabling high‐resolution mapping of Young's modulus and energy dissipation. This approach allowed us to correlate changes in mechanical performance with structural transitions and electronic properties, offering a multidimensional perspective on the MOF‐to‐porous oxide transformation, a key innovation of this study.

## 3 Microstructural and Nanoscale Morphological Evolution

The morphological and structural evolution of the Cu‐BTC metal‐organic framework and its thermally derived porous oxide phases were systematically investigated through an integrative characterization strategy combining high‐resolution SEM with bimodal AFM phase and topography imaging. This correlative approach provided unprecedented nanoscale insights into the dimensional and compositional transformations induced by progressive calcination, revealing intricate structural changes beyond the scope of conventional imaging techniques.

**FIGURE 2:**
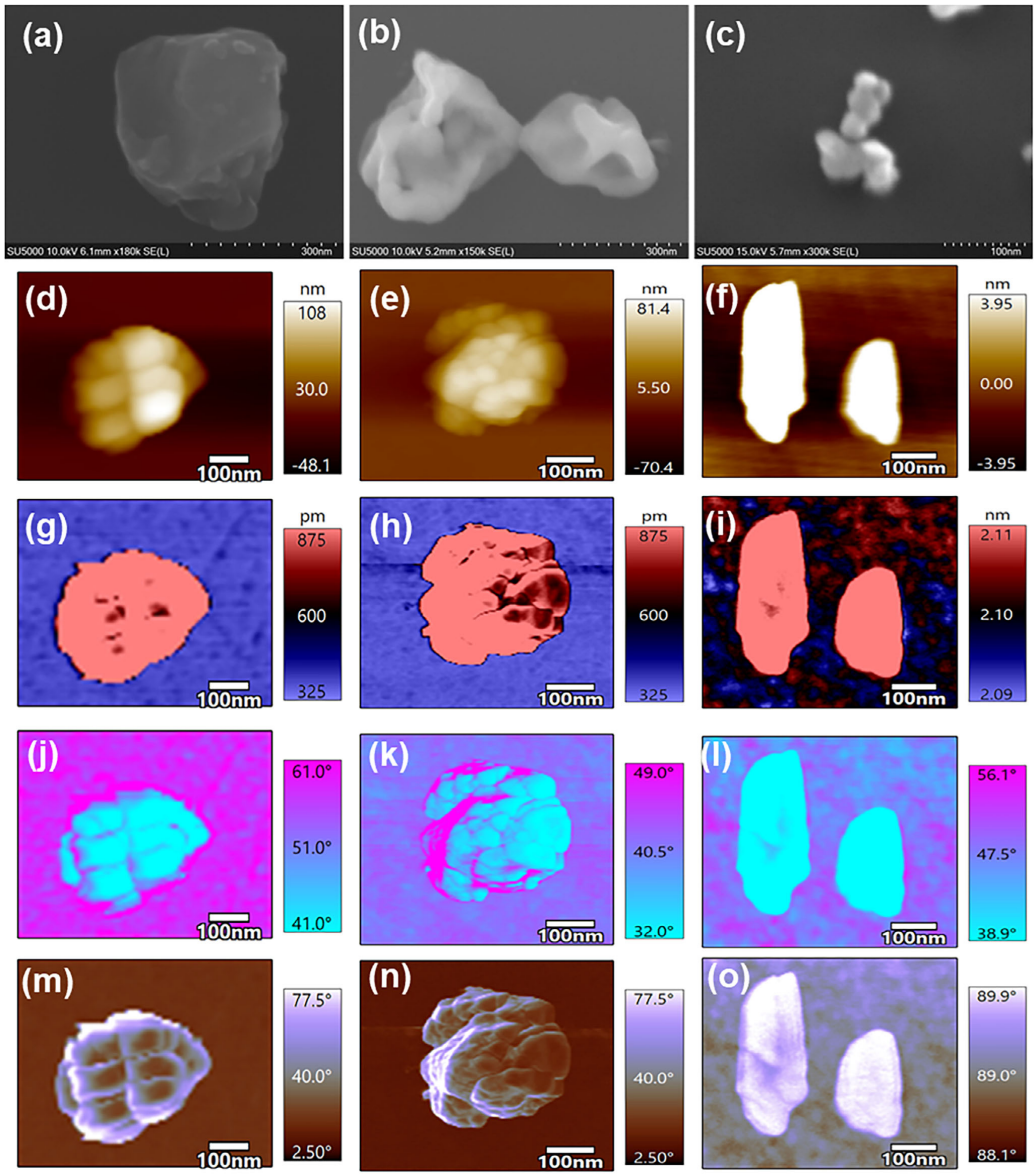
FESEM images of (a) Cu‐BTC MOF, (b) Cu‐MOF calcined at 300°C, (c) Cu‐MOF calcined at 500°C; Bimodal dual ac AM‐AM AFM topography images of (d) Cu‐BTC MOF, (e) Cu‐MOF calcined at 300°C, (f) Cu‐MOF calcined at 500°C; second amplitude images of (g) Cu‐BTC MOF, (h) Cu‐MOF calcined at 300°C, (i) Cu‐MOF calcined at 500°C; first phase images of (j) Cu‐BTC MOF, (k) Cu‐MOF calcined at 300°C, (l) Cu‐MOF calcined at 500°C; second phase images of (m) Cu‐BTC MOF, (n) Cu‐MOF calcined at 300°C, (o) Cu‐MOF calcined at 500°C. Correlative FESEM and bimodal AFM analyses reveal a thermally induced transformation of Cu‐BTC from a porous, heterogeneous MOF (∼400 nm) to smaller, denser oxide structures (∼140 nm at 300°C and ∼85 nm at 500°C), with reduced roughness and complexity; while SEM shows particle size reduction, AFM phase and amplitude imaging capture the transition from porous framework to a Cu_2_O/CuO intermediate and finally to a porous yet more homogeneous CuO phase.

Initial SEM analysis provides foundational morphological data, capturing the clear trend of particle size reduction as a direct consequence of elevated thermal treatment (Figure 2a–c). Pristine Cu‐BTC samples exhibited well‐defined crystalline particles with lateral dimensions near 400 nm. Following calcination, these dimensions contracted markedly to approximately 140 nm at 300°C and further to 85 nm at 500°C, indicating substantial densification and framework collapse. Despite the clarity of size diminution, SEM topography alone failed to resolve the subtle porous architecture and compositional heterogeneity inherently embedded within the MOF precursor, underscoring the need for complementary nanoscale phase‐sensitive imaging.

To overcome this limitation, bimodal AFM was used to record surface topography and phase images (Figure 2d–o) simultaneously, allowing detailed analysis of mechanical and compositional variations at the nanoscale. The topography images obtained from the bimodal dual ac AM‐AM for the pristine Cu‐BTC MOF, Cu‐MOF calcined at 300°C, and Cu‐MOF calcined at 500°C are shown in Figure 2d–f. Quantitative nanoscale topographical metrics extracted from bimodal AFM show the details of thermally induced progression. The lateral particle dimensions followed a consistent downward trend (Figure 2d–i) from ∼400 nm (pristine MOF) to ∼140 nm (300°C) and finally ∼85 nm (500°C), paralleled by a systematic decrease in surface roughness (as summarized in Table S1). This trend highlights the gradual loss of surface complexity and porosity as the MOF framework decomposes and densifies into copper oxide phases.

The pristine Cu‐BTC framework exhibited strong phase contrast heterogeneity, reflecting its porous structure composed of flexible organic linkers and rigid copper metal nodes. This heterogeneity appeared as distinct domains in the phase images (Figure 2j,m), highlighting the coexistence of different material components that underpin the MOF's high surface area and functional performance. The porous nature of Cu‐BTC is clearly visible in the second‐mode phase image (Figure 2m).

Thermal calcination at 300°C initiated a pivotal transformation characterized by the progressive volatilization of organic linkers. Phase imaging (Figure 2k,n) revealed a heterogeneous intermediate state where residual porosity coexisted with emerging metal oxide phases, specifically, a composite of cuprous oxide (Cu_2_O) and cupric oxide (CuO). This transitional phase retained discernible porosity, reflected in moderate phase contrast variations and partial maintenance of nanoscale surface roughness. These results indicate a partial but well‐defined restructuring of the MOF framework, revealing the transition from a flexible, porous organic–inorganic hybrid to a phase dominated by oxide components.

Further calcination at 500°C culminated in the formation of a porous CuO phase. While the topography image (Figure 2f) at this stage displayed markedly homogenized contrast, consistent with the absence or significant reduction of organic moieties and the establishment of a dense, crystalline oxide structure, the bimodal phase images (Figure 2l,o) as well as the second mode amplitude image (Figure 2i) clearly show the porous structure of P‐CuO. Concurrently, topographical maps corroborated this transformation by demonstrating further particle size reduction and significant surface smoothing down to 1.1 ± 0.3 nm (as shown in Table S1 for roughness measurements), consistent with the collapse of porous frameworks and the generation of a rigid metal oxide lattice.

Collectively, these multimodal analyses elucidate the critical role of organic linkers in maintaining MOF porosity and reveal the mechanistic pathway by which thermal treatment drives framework collapse and phase evolution. The unique sensitivity of bimodal phase imaging to mechanical and compositional heterogeneity provides unparalleled insight into these subtle yet essential transformations, insights that are obscured in conventional SEM or topographical‐only AFM studies. This integrative characterization thus offers a comprehensive understanding of the MOF‐to‐porous oxide conversion, paving the way for rational design and controlled synthesis of advanced porous materials with tailored structural and functional properties.

## 4 Phase Composition and Surface Chemistry Characterization via XRD and XPS

Following the elucidation of morphological and structural evolution, it is essential to interrogate the underlying phase composition and chemical states that govern the observed physical transformations. To this end, XRD and XPS analyses were systematically employed, bridging the gap between nanoscale morphology and material identity at both bulk and surface levels.

### 4.1 XPS Analysis: Surface Chemical States and Elemental Composition

XPS was used to examine the surface chemical composition and oxidation states of key elements in the Cu‐BTC precursor and its thermally transformed derivatives. The survey spectra (Figure S2) clearly confirmed the presence of carbon (C), oxygen (O), and copper (Cu) as the main elements, with no detectable impurities, confirming the high purity of the samples.

Quantitative compositional analysis (as summarized in Table S2 and Figure S3) revealed a progressive decrease in surface carbon content with increasing calcination temperature, descending markedly from 51.05% in the pristine Cu‐BTC to 16.88% and 15.98% at 300°C and 500°C, respectively. This decline corroborates the thermal decomposition of organic linkers during calcination.

**FIGURE 3:**
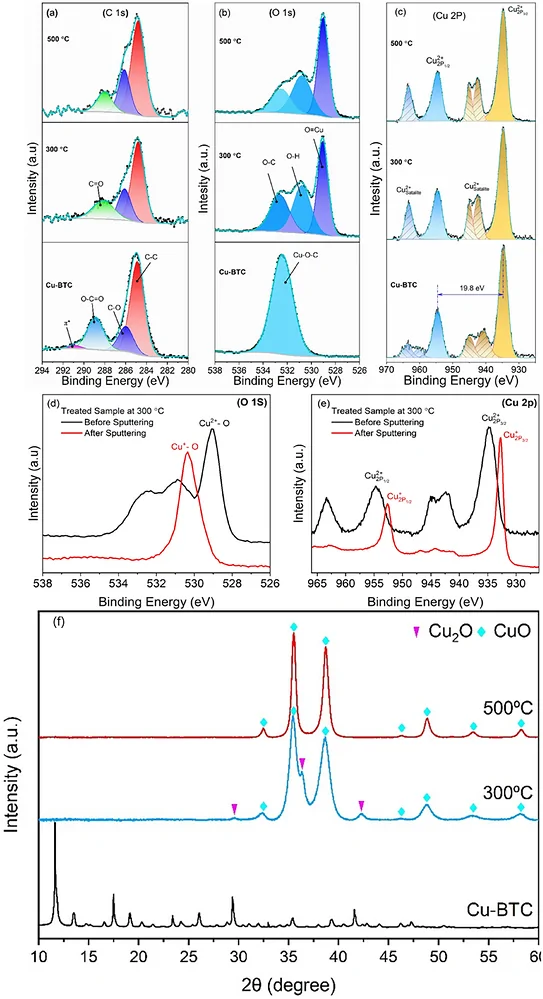
High‐resolution XPS spectra of (a) C 1s, (b) O 1s, and (c) Cu 2p; (d) and (e) High‐resolution spectrum of O 1s and Cu 2p after applying sputtering for profile depth for Cu BTC MOF annealed at 300°C. (f) XRD patterns of heat‐treated Cu‐BTC at different temperatures. XPS confirms high purity and reveals thermally induced transformation of Cu‐BTC, with significant carbon loss from linker decomposition and the emergence of oxide species; high‐resolution spectra indicate conversion to CuO with dominant Cu(II) states, while depth profiling uncovers a CuO‐rich surface over a Cu_2_O subsurface, consistent with XRD results.

High‐resolution XPS spectra of C 1s, O 1s, and Cu 2p were further deconvoluted to elucidate chemical bonding variations (Figure 3a–c). The Lorentzian‐Gaussian fitting of the C 1s spectrum of pristine Cu‐BTC (Figure 3a, peak fitting results are summarized in Table S3) revealed three main peaks plus a satellite one. The exhibited characteristic peaks at 284.8 eV (C─C), 286.1 eV (C─O), and 288.9 eV (O═C─O) [19], alongside a π–π* satellite at ∼291 eV, are indicative of the aromatic organic ligands inherent to the MOF structure [20]. Thermal treatment at 300°C and 500°C led to the disappearance of the satellite peak and the carboxylate‐related O═C─O peak, evidencing the loss of aromatic and carboxyl functional groups and the transformation into copper oxide phases. The treated samples displayed peaks at 284.8, 286.1, and 288.1 eV, assignable to C─C, C═O, and C═O species, respectively [21], reflecting residual surface carbon species. The change in the carbon species during calcination was supported by the compositional analysis as summarized in Table S3. The O 1s spectra (Figure 3b) provided further insight into oxygen chemical states. For Cu‐BTC, a dominant peak at 532.6 eV was attributed to Cu─O─C linkages, consistent with carboxylate groups within the MOF framework [19, 22, 23]. Upon calcination, the spectra evolved into three distinct peaks at ∼529, 530.7, and 532.6 eV, corresponding to lattice oxygen (O^2^^−^) in CuO, surface hydroxyl groups (O─H), and adsorbed oxygen species (O─C), respectively, adsorbed onto the surface of P‐CuO*_x_* [24]. The increasing dominance of the lattice oxygen peak with temperature (summarized peak fitting data is shown in Table S4) shows an increase in the proportion of lattice oxygen (O═Cu) at 500°C aligned with the progressive formation of CuO.

The Cu 2p spectra (Figure 3c) further illuminated the copper oxidation states. All samples exhibited the characteristic Cu 2p_3_/_2_ and Cu 2p_1_/_2_ peaks at approximately 934.8 and 954.6 eV, consistent with the data recorded in literature for Cu 2p [25, 26], with a consistent spin‐orbit splitting of 19.8 eV, the same as the standard Cu 2p spectrum reported in literature [27].

The peak shape was fitted by a Gaussian/Lorentzian line‐shape (GL30, which was modified by an exponential blend to better match the observed data). The results of peak fitting revealed convoluted peaks that are attributed to the oxidation state of Cu (II) for all samples. Additionally, the presence of the satellite peaks indicates a partially filled 3d^9^ shell due to the higher oxidation state of Cu (II) [26, 27, 28]. It was noticed that for samples at 300°C and 500°C, these satellite peaks become strong and are positioned at 942.4, 945, and 963.3 eV. The strong satellite peaks provided evidence of Cu (II) oxidation state, indicating the existence of CuO phase and negating the likelihood of a Cu_2_O phase being present. Interestingly, despite the XRD study (Figure 3f) indicating coexistence of CuO and Cu_2_O phases at 300°C, the XPS spectra primarily reflected Cu(II) states, suggesting that the CuO phase preferentially resides at the surface while Cu_2_O is buried beneath. This surface dominance of CuO was further validated via depth profiling (Figure 3d–e), where incremental sputtering removed the CuO outer layer, revealing underlying Cu_2_O characterized by the disappearance of satellite peaks and the appearance of Cu(I) Cu 2p peaks at 932.7 and 952.6 eV, along with an O 1s peak at 530.3 eV. These findings confirm a stratified oxide structure, with a CuO‐enriched surface encapsulating a Cu_2_O core, consistent with established literature [29, 30].

It should be noted that the Cu 2p region alone cannot unambiguously quantify the relative Cu(I)/Cu(II) surface fractions because of the partial overlap between Cu_2_O and CuO features and the absence of shake‐up satellites for Cu(I). Therefore, in the absence of Cu LMM Auger or Cu 3p/Cu 3s spectra, the proposed CuO‐enriched surface and Cu_2_O‐rich subsurface/core structure is interpreted from the combined evidence of XRD (discussed in the next section), O 1s/Cu 2p XPS spectra, and Ar^+^ ion sputtering followed by XPS analysis, rather than solely from Al Kα Cu 2p XPS.

### 4.2 XRD Analysis: Crystallographic Phase Evolution

Figure 3f illustrates the XRD patterns collected over a 2θ range of 10^°^–60°, highlighting the effects of temperature on the crystallographic structure of Cu‐BTC. At the calcination temperature of 300°C in an air environment, the Cu‐BTC transformed into a mixture of copper oxides (CuO and Cu_2_O). The predominant phase observed is CuO, with the presence of Cu_2_O indicated by diffraction peaks at 2θ positions of 29.64°, 36.52°, and 42.42°, corresponding to the (110), (111), and (200) planes of Cu_2_O, respectively. These peaks showed strong alignment in position and intensity with ICDD Card No. 01‐077‐0199.

Although calcination was performed under closed conditions in the muffle furnace, the furnace atmosphere was not intentionally oxygen‐limited. The small precursor mass and thin powder layer used in this work allowed direct exposure to the surrounding atmosphere. The formation of Cu_2_O at 300°C is therefore attributed to an intermediate oxidation stage during Cu‐BTC decomposition, consistent with the temperature and heating‐rate‐dependent transformation pathway reported previously [15]. The availability of O_2_ in the muffle furnace was sufficient to avoid partial reduction, which was confirmed and reported in our earlier communication [15].

When the calcination temperature is raised to 500°C, the XRD patterns reflect a phase conversion, resulting in P‐CuO. The characteristic peaks, now present at 2θ values of 32.5°, 35.4°, 38.7°, 46.3°, 48.7°, 51.3°, 53.4°, and 58.3°, align with the (110), (002), (111), (−112), (−202), (112), (020), and (202) crystallographic planes of CuO. This phase purity at 500°C underscores the temperature‐dependent transformation of Cu‐BTC into a single‐phase CuO, confirmed by its consistency with the card no. 00‐005‐0661 for CuO. The emergence of these well‐defined peaks signifies the consolidation and crystallization of copper oxide into a thermodynamically stable, single‐phase oxide structure.

## 5 Nanomechanical Properties via Bimodal AFM

**FIGURE 4:**
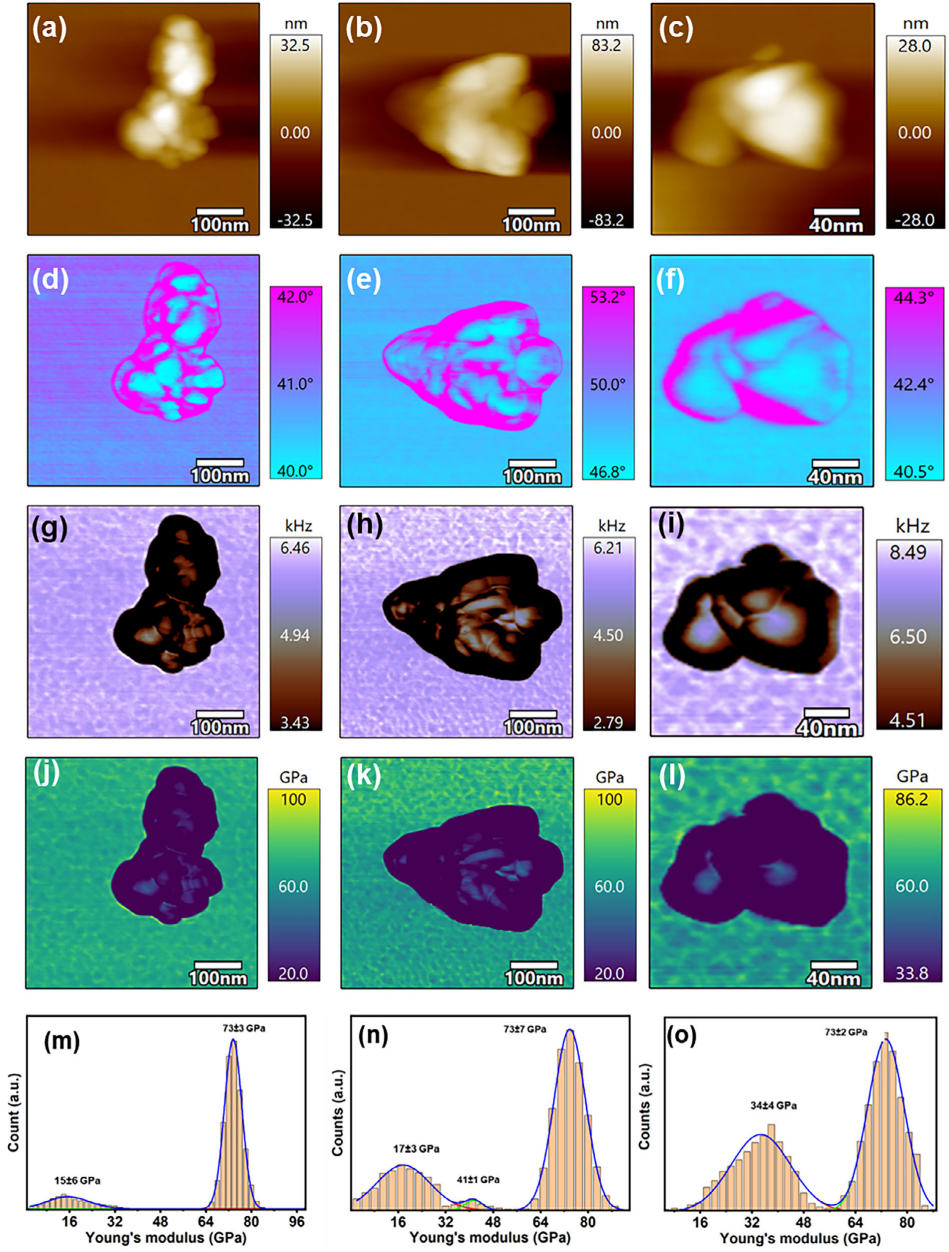
Bimodal AM‐FM AFM topography, phase, frequency shift, and Young's modulus images of (a, d, g, and j) Cu‐BTC MOF, (b, e, h, and k) Cu‐MOF calcined at 300°C, (c, f, i, and l) Cu‐MOF calcined at 500°C, respectively. The histograms from the Young's modulus maps and the corresponding fitting for (m) Cu‐BTC MOF, (n) Cu‐MOF calcined at 300°C, (o) Cu‐MOF calcined at 500°C. Bimodal AM–FM AFM mapping reveals a non‐monotonic evolution of nanomechanical stiffness, with pristine Cu‐BTC showing low modulus (∼15 GPa), a peak stiffness at 300°C (∼41 GPa) due to heterogeneous Cu_2_O‐rich mesoporous scaffolds, and a reduced modulus at 500°C (∼34 GPa) associated with structurally coarsened but chemically homogeneous CuO.

**FIGURE 5:**
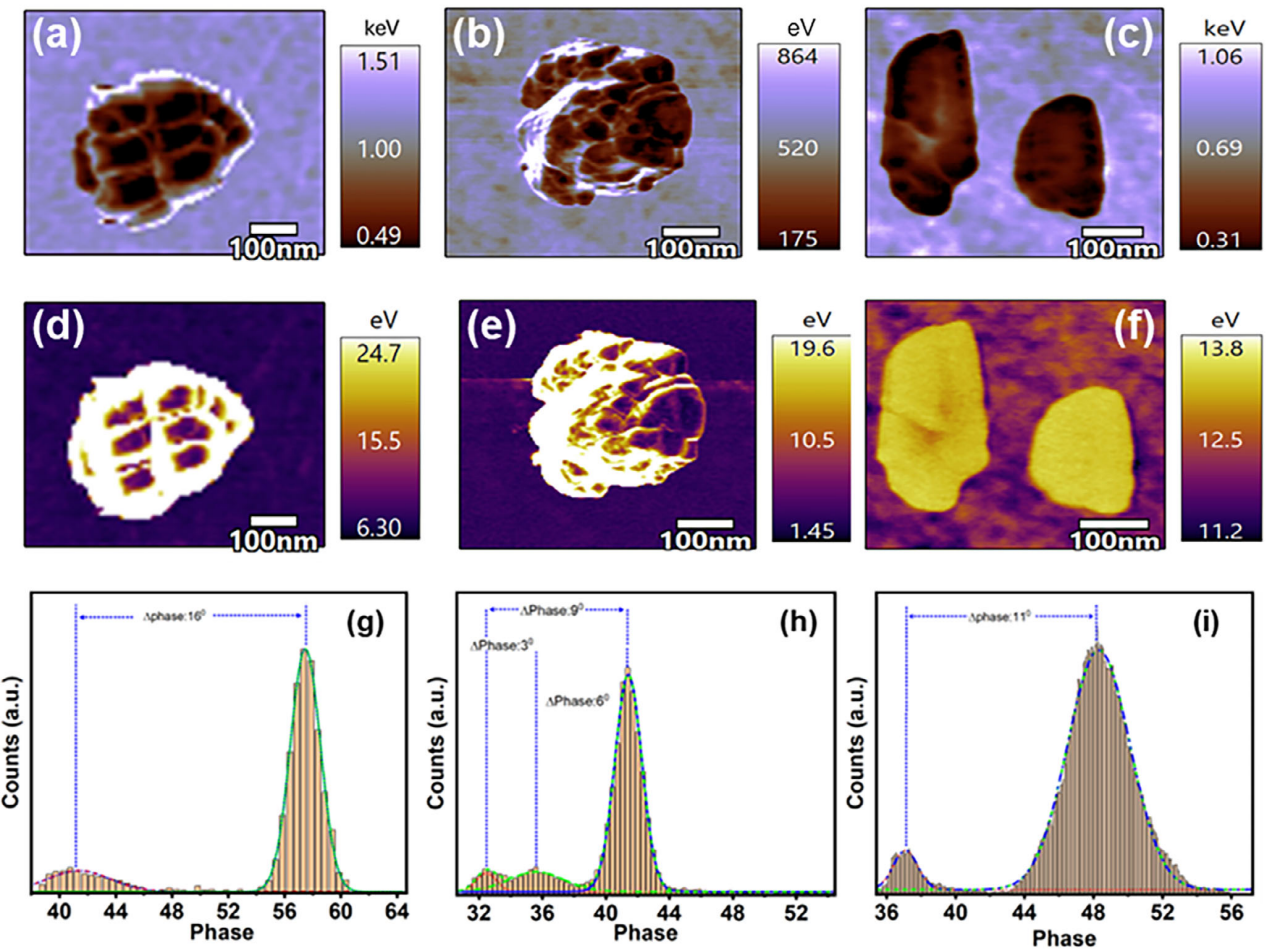
Dissipation maps obtained from the first and second eigenmodes for (a, d) Cu‐BTC MOF, (b, e) Cu‐MOF calcined at 300°C, (c, f) Cu‐MOF calcined at 500°C. The Histograms of the first phases and the corresponding Gaussian fits and phase differences for (g) Cu‐BTC MOF, (h) Cu‐MOF calcined at 300°C, and (i) Cu‐MOF calcined at 500°C. Energy dissipation and phase imaging show mode‐ and composition‐dependent damping, with high dissipation in porous Cu‐BTC, reduced dissipation at 300°C, and a slight increase at 500°C due to homogeneous CuO and interfacial losses.

Following the structural and chemical evolution of Cu‐BTC under thermal treatment, we investigated the impact of these changes on the material's nanomechanical behavior using bimodal atomic force microscopy. Bimodal AFM, specifically amplitude modulation–amplitude modulation (AM–AM) and amplitude modulation–frequency modulation (AM–FM) techniques, enables simultaneous mapping of surface topography, mechanical stiffness, and energy dissipation at the nanoscale [31, 32, 33]. This multimodal approach is particularly advantageous for evaluating materials like Cu‐BTC that undergo profound chemical transformations upon calcination, offering insight into the relationship between structure, porosity, and mechanical performance. In the AM–FM modality (Figure 4), stiffness was evaluated by extracting Young's modulus values across thermally treated samples. The as‐prepared Cu‐BTC exhibited two distinct mechanical domains: the stiffer silicon oxide substrate (73 ± 3 GPa) and the softer Cu‐BTC framework (15 ± 6 GPa, Figure 4m), consistent with its porous, organic–inorganic hybrid nature (Figures 4 and 5).

The nanomechanical mapping reveals a clear but non‐monotonic evolution of stiffness with calcination temperature. At 300°C, the Cu_2_O‐rich intermediate exhibits regions with a high apparent Young's modulus of 41 ± 1 GPa (Figure 4n), alongside domains with significantly lower stiffness. Upon further calcination to 500°C, the material transforms into a chemically homogeneous CuO phase, with a reduced average modulus of 34 ± 4 GPa (Figure 4o). At first glance, this trend appears counterintuitive, as bulk CuO is intrinsically stiffer than Cu_2_O. This apparent contradiction is resolved by considering nanoscale heterogeneity and temperature‐induced microstructural evolution.

At 300°C, the material is in a transitional state. Most organic linkers have decomposed, but remnants of the MOF‐derived porous architecture persist together with newly formed Cu_2_O crystallites and early‐stage CuO layers. AFM nanomechanical maps directly capture this heterogeneity, revealing a patchwork of stiff domains (∼41 GPa) embedded within mechanically weaker regions. The stiff domains originate from a Cu_2_O‐based scaffold that inherits the finely connected mesoporous network of the parent MOF. To evaluate the porosity and surface area of the Cu‐BTC, Cu‐BTC calcined at 300°C, and Cu‐BTC calcined at 500°C samples, a comprehensive Brunauer–Emmett–Teller (BET) analysis was performed (detailed description provided in the Section S1). The corresponding isotherms, pore volume, and pore width are presented in Figure S4, while the calculated surface area, pore width, and total pore volume of all samples are summarized in Table S5. The BET analysis confirms the presence of this templated structure, showing a reduced but still substantial surface area (33 m^2^ g^−^^1^) and pore sizes concentrated in the mesoporous range (∼12 nm). This interconnected mesoporosity enables efficient stress redistribution, locally enhancing the effective modulus.

In contrast, the low‐modulus regions at 300°C are attributed to residual organic species, amorphous carbonaceous fragments, and mechanically weak interfacial zones between evolving oxide phases. These softer domains reflect the incomplete and spatially heterogeneous nature of the thermal transformation. As a result, the 300°C sample cannot be considered mechanically uniform, but rather a composite consisting of locally stiff Cu_2_O‐rich scaffolds embedded within softer, partially collapsed regions.

Following calcination to 500°C, phase conversion is complete, and AFM maps indicate a chemically homogeneous CuO material. However, this chemical uniformity is accompanied by pronounced microstructural degradation. BET measurements (Figure S4 and Table S5) reveal a three‐fold reduction in surface area (to 11 m^2^ g^−^^1^) and an increase in average pore diameter to ∼22 nm, indicative of pore coarsening and partial collapse of the templated framework. SEM and AFM analyses (Figure 2) further show particle shrinkage (from ∼140 to ∼85 nm) and reduced surface roughness (Table S1), consistent with sintering and densification. Additionally, the crystallographic transition from Cu_2_O to CuO involves lattice expansion and symmetry changes that introduce local stresses and weaken interparticle connections. Together, these effects reduce the load‐bearing efficiency of the porous network, leading to a lower effective modulus despite the higher intrinsic stiffness of CuO.

Taken together, these observations establish a clear structure–property relationship. The anomalously high modulus at 300°C arises from well‐connected mesoporous Cu_2_O‐rich scaffolds templated from the parent MOF, while the softer regions reflect incomplete framework collapse and residual organic content. At 500°C, although the oxide phase is intrinsically stiffer, the loss of fine mesoporosity, pore enlargement, and transformation‐induced stresses degrade the mechanical integrity of the structure. Thus, the effective mechanical response of MOF‐derived copper oxides is governed primarily by pore architecture and nanoscale heterogeneity rather than by bulk phase composition alone.

To further elucidate the chemical origins of this mechanical evolution, we analyzed the atomic composition obtained from XPS survey spectra (Figure S2), with the relative atomic percentages of carbon, oxygen, and copper summarized in Figure S3. The data confirm a transition from the linker‐dominated pristine MOF (C ≈ 51%) to an inorganic‐rich material upon calcination. Notably, a substantial carbon fraction (∼16%–17%) persists in both the 300°C and 500°C samples, indicating that these materials are oxide–carbon nanocomposites rather than pure oxides. This insight provides an important framework for interpreting the mechanical data.

The highest Young's modulus observed at 300°C (peak 2, ∼41 GPa, Figure 4n) coincides with the maximum copper concentration (∼45%), suggesting an optimal composite architecture in which a mechanically robust, copper‐rich inorganic scaffold is reinforced by a residual carbon matrix. Upon further heating to 500°C, the copper content decreases slightly (∼43%) while the oxygen‐to‐copper ratio increases, consistent with further oxidation toward CuO. This compositional and microstructural evolution correlates with the observed reduction in stiffness (∼34 GPa, Figure 4o), indicating that the 300°C nanocomposite structure provides superior mechanical performance compared with the more fully oxidized but structurally coarsened material obtained at 500°C.

The analysis of energy dissipation, extracted from AM–AM mode in the repulsive regime (Figure 5), revealed critical differences among the samples, reflecting their evolving nanomechanical energy loss pathways. As seen, all samples exhibit a reversal in dissipation contrast between the MOF (Cu‐BTC and its thermally annealed derivatives) and the substrate across the first and second flexural modes.

Specifically, in the first eigenmode, the energy dissipation values for Cu‐BTC, as well as for the Cu‐BTC samples calcined at 300°C and 500°C, are lower than those of the substrate (Figure 5a–c). This suggests reduced energy loss through tip‐sample interactions at low‐frequency oscillations, possibly due to the compliant and porous nature of the MOF framework, which absorbs less energy under small deformation amplitudes. In this regime, energy loss is likely dominated by elastic interactions or interfacial damping. In contrast, in the second eigenmode, where the tip oscillates at higher frequencies and experiences increased stiffness sensitivity, the dissipation from all MOF‐based samples exceeds that of the substrate (Figure 5d–f). This heightened energy loss is indicative of frequency‐dependent dissipative mechanisms such as internal friction from structural heterogeneities or interfacial effects resulting from partially decomposed organic linkers or the onset of crystalline oxide phases. The higher mode likely couples more strongly to deeper mechanical and structural fluctuations within the MOF, especially in partially converted or collapsed frameworks. This mode‐dependent reversal in dissipation behavior highlights the complex, multiscale energy loss processes occurring within the MOF‐to‐oxide transformation and underscores the value of multi‐frequency AFM for disentangling them. It also suggests that while the low‐frequency mechanical response remains dominated by bulk compliance, higher‐frequency responses are increasingly governed by localized heterogeneities, phase boundaries, and dissipative channels introduced during thermal annealing.

Furthermore, we analyzed and compared the phase shifts recorded in AM–AM mode between Cu‐BTC, Cu‐BTC samples calcined at 300°C and 500°C, and the substrate (Figure 5g–i). Phase shift in this context reflects the dissipative component of the tip‐sample interaction, providing insights into energy loss mechanisms at the nanoscale. An interesting observation, consistent with the trends seen in the Young's modulus measurements, is the intermediate phase response of the sample calcined at 300°C, which contains a mixed composition of Cu_2_O and CuO. This mixture arises from partial decomposition of the Cu‐BTC framework and incomplete oxidation of copper species at this temperature.

As illustrated in Figure 5g, the pristine Cu‐BTC sample exhibits a relatively high phase shift of approximately 16°, indicative of significant energy dissipation due to its porous structure and the presence of dynamic interactions such as interfacial friction within the MOF network.

In contrast, the phase shifts observed for the mixed‐phase sample annealed at 300 °C are lower, around 6° for regions dominated by Cu_2_O and 9° for those richer in CuO (Figure 5h). This reduction in phase shifts reflects the increased mechanical stiffness as reported in our stiffness measurement and reduced internal friction resulting from the partial transformation of the MOF into more crystalline and compact oxide domains. For the fully converted CuO sample annealed at 500 °C, the phase shift increases slightly to around 11° (Figure 5i), likely due to the more homogeneous and crystalline nature of the final oxide, which may exhibit enhanced interfacial energy losses under oscillatory loading despite its higher stiffness. These variations in phase shift across the different thermal states of the MOF samples reveal how the mechanical damping behavior evolves with structural and compositional changes. Specifically, the decreasing phase shift from Cu‐BTC to the 300°C sample indicates a transition from a dissipative, porous framework to a more rigid, mixed‐phase oxide, followed by a moderate increase in dissipation in the final CuO state due to its dense polycrystalline texture.

Overall, the combined bimodal stiffness and dissipation measurements provide a comprehensive picture of the nanomechanical evolution of Cu‐BTC upon thermal annealing. The data clearly illustrate how the progressive transformation from a softer, porous metal‐organic framework to a rigid oxide phase systematically alters both elastic and dissipative material responses.

## 6 Structure–Property Correlations via PCA and Multivariate Analysis

**FIGURE 6:**
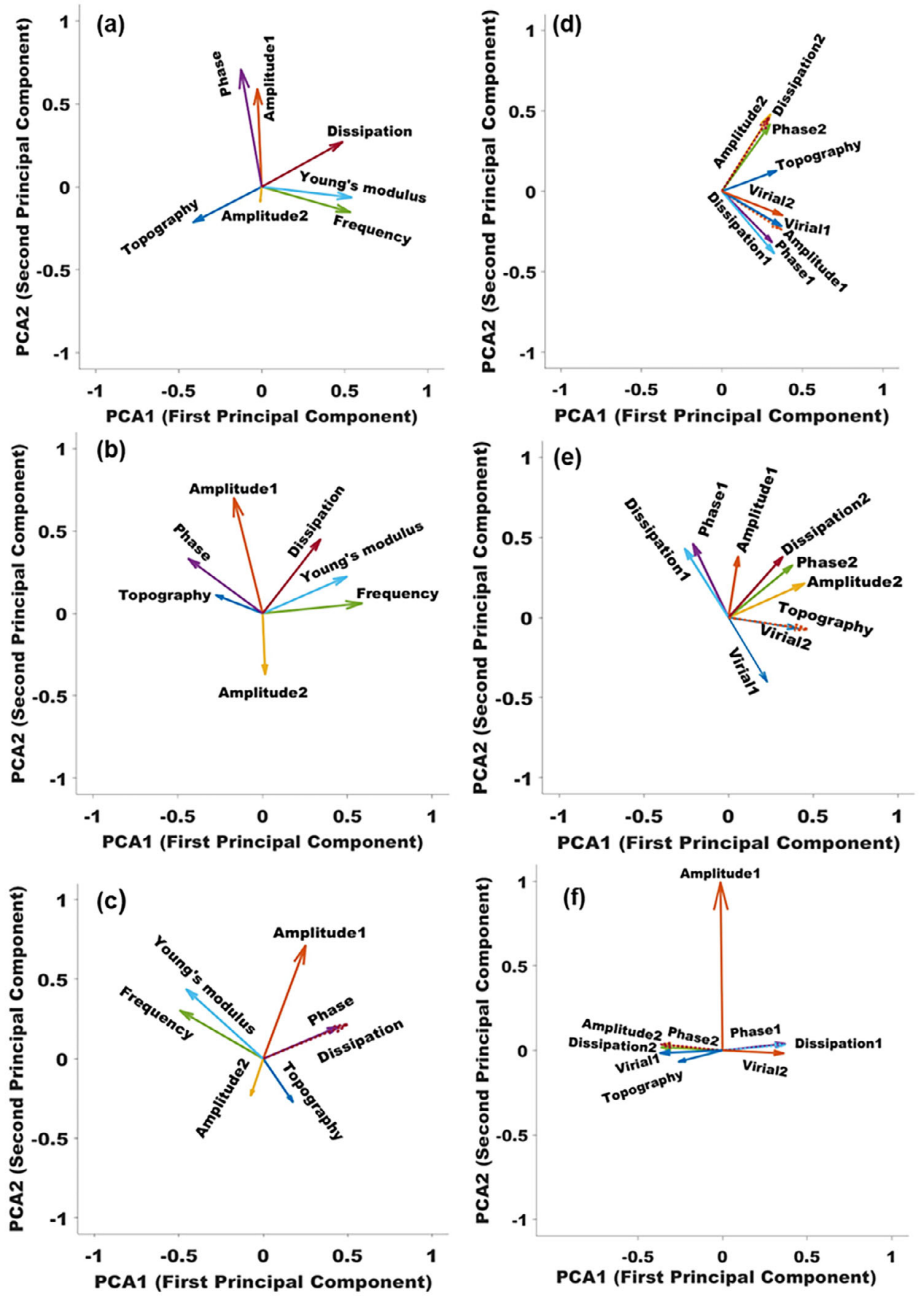
PCA loading plots of (a) Cu‐BTC MOF, (b) Cu‐MOF calcined at 300°C, (c) Cu‐MOF calcined at 500°C, obtained from AM‐FM AFM and PCA analysis from the bimodal dual‐AC AFM analysis of (d) Cu‐BTC MOF, (e) Cu‐MOF calcined at 300°C, and (f) Cu‐MOF calcined at 500°C. PCA shows a transition from homogeneous porous Cu‐BTC to a coupled heterogeneous state at 300°C, and finally to uniform CuO with distinct stiffness and dissipation behavior.

To elucidate the structural and mechanical transformation from Cu‐BTC to porous CuO, we applied PCA to datasets acquired using AM–FM and AM–AM AFM modalities. PCA reveals how mechanical, topographical, and dissipative observables evolve during thermal conversion, complementing stiffness and dissipation measurements and providing mechanistic insight into nanoscale phase evolution (Figure 6).

In AM–FM PCA analysis, for pristine Cu‐BTC (Figure 6a), two main clusters emerge: Young's modulus, frequency shift, and dissipation (stiffness and energy loss), and phase with amplitude 1. Amplitude 2 and topography are uncorrelated, indicating a mechanically homogeneous but structurally porous framework where elasticity dominates tip–sample interactions. After annealing at 300°C (Figure 6b), the formation of a mixed Cu_2_O/CuO phase leads to clustering of phase, amplitude 1, and topography, reflecting stronger coupling between morphology and viscoelastic response, while modulus, dissipation, and frequency shift remain correlated. Amplitude 2 stays decoupled, suggesting sensitivity to larger or localized structural changes. At 500°C (CuO, Figure 6c), the system becomes mechanically uniform: modulus and frequency shift define a stiffness cluster, dissipation and phase form an energy‐loss cluster, amplitude 1 bridges both, and topography and amplitude 2 are fully uncorrelated.

In AM–AM PCA analysis, for Cu‐BTC (Figure 6d), two dominant clusters appear: a second‐mode group (amplitude 2, phase 2, dissipation 2) sensitive to subsurface interactions and a first‐mode group (amplitude 1, phase 1, dissipation 1, virials), indicating dual dissipation mechanisms. Topography remains uncoupled. At 300°C (Figure 6e), the second‐mode cluster persists, while topography and virial 2 form a separate group, showing increasing morphological influence. First‐mode observables remain tightly correlated, consistent with increased stiffness (∼41 GPa) and reduced low‐frequency dissipation. In fully converted CuO (500°C, Figure 6f), PCA shows stronger coupling between topography and dissipation, consistent with nanograin boundaries and enhanced friction in porous CuO, while amplitude 1 remains distinct as a transitional observable. Full multivariate results and corresponding analysis are provided in Section S2 and Figures S5–S7.

## 7 UV–Vis Measurements and Ultraviolet Photoelectron Spectroscopy (UPS) Study

The evolution of the optical properties during the thermal transformation of Cu‐BTC to nanoporous copper oxides was studied using UV–Vis–NIR spectroscopy. The absorptance and scattering characteristics of pristine Cu‐BTC and its calcined derivatives (P‐CuO*_x_*) at different temperatures (Figure S8) provide insight into the change in electronic structure and light absorption capacity, which are crucial for applications in solar energy conversion. Figure S8a,b presents the measured transmittance (%T) and combined transmittance plus scattering (%T+S) for 0.02 wt% fluid dispersions of Cu‐BTC and P‐CuO*_x_* in ethanol. The absorption and scattering coefficients were determined by measuring the transmittance and the combined transmittance plus scattering of light passing through a 10 mm optical path length of the P‐CuO*_x_*/ethanol fluid dispersion, following established methods reported in the literature [34, 35]. The resulting plots are presented in Figure S8c,d. Detailed calculations of the optical properties, particularly the absorption and scattering coefficients, are provided in Section S3 and Figure S9. These coefficients enable differentiation between light attenuation caused by true absorption and that arising from scattering within the dispersion. These coefficients provided insight into how effectively the fluid dispersions absorb and scatter light across a range of wavelengths, from 200 to 1000 nm. Cu‐BTC/ethanol exhibited the weakest absorption, especially below 600 nm. As seen in Figure S8c, the absorption coefficient dropped to nearly zero at around 550 nm and remained low between 550 and 350 nm before increasing at shorter wavelengths. This behavior is attributed to strong scattering in this region, as indicated by the transmittance plus scattering spectrum in Figure S8b, where values approached nearly 100%, confirming minimal absorption. In contrast, P‐CuO*_x_*/ethanol fluid dispersions displayed significantly improved absorption characteristics across a wide range of wavelengths. The absorption coefficients increased at shorter wavelengths, while scattering effects became more prominent at longer wavelengths (Figure S8c,d). The dispersion of the 300°C sample, representing a transitional phase composed of Cu_2_O and CuO, showed a moderate improvement in absorption compared to the pristine Cu‐BTC. Meanwhile, the 500°C sample, composed predominantly of crystalline CuO, demonstrated a strong and highest absorption profile across the spectrum. This enhancement is attributed to the successful transformation into a semiconducting oxide phase with a well‐developed porous structure, facilitating stronger light‐matter interactions.

To elucidate the photophysical properties of Cu‐BTC and the Cu‐BTC‐derived P‐CuO*_x_* samples calcined at 300°C and 500°C, the optical bandgap energies were determined from Tauc plot analysis of the UV–Vis–NIR absorption spectra. A detailed discussion of the bandgap estimation, together with the corresponding Tauc plots are provided in Section S4 and Figure S10. A comparison of the bandgap values obtained experimentally from UV–Vis–NIR spectroscopy, predicted by DFT calculations, and reported in the literature for Cu‐BTC and chemically synthesized CuO*_x_* materials is summarized in Table 1.

**TABLE 1:** Comparison of experimental and computational bandgap energies obtained in this study with literature‐reported values for pristine Cu‐BTC and CuO [36, 42, 43].

Source of bandgap energy	Pristine Cu‐BTC	CuO
Experimental bandgap value (this work)	4.05 eV	1.56 eV
Computational bandgap value (this work)	3.60 eV	1.42 eV
Experimental bandgap value (literature)	3.68 eV	1.44 eV

To gain deeper insight into the electronic band structure of these materials, ultraviolet photoelectron spectroscopy (UPS) was employed to determine key electronic parameters such as the work function, valence band maximum (VBM), and conduction band minimum (CBM) of Cu‐BTC and the Cu‐BTC‐derived P‐CuO*_x_* samples calcined at 300°C and 500°C. Furthermore, to probe depth‐dependent electronic characteristics, UPS measurements of the sample calcined at 300°C were performed both before and after sputtering, which enabled the differentiation between surface and subsurface electronic structures.

The UPS spectra presented in Figure S12 highlight the secondary electron cut‐off energy (*E*_Cut‐off_) and valence band onset energy (*E*_On‐set_) of these materials. Combining the optical bandgap energies derived from Tauc plot analysis with the UPS‐determined electronic parameters, comprehensive energy band diagrams were constructed for all samples, as shown in Figure S13. A detailed description of the work function determination, band edge calculations, and energy band diagram construction for Cu‐BTC and the Cu‐BTC‐derived P‐CuO*_x_* samples calcined at 300°C and 500°C is provided in Section S5.

## 8 Mechanistic Model of MOF‐to‐Oxide Conversion

The thermally driven conversion of Cu‐BTC, a prototypical copper‐based MOF, into porous crystalline CuO proceeds through a multistage cascade of strongly coupled structural, electronic, and nanomechanical transformations. Rather than a monotonic progression towards a mechanically superior oxide, the system traverses a non‐equilibrium intermediate state in which pore architecture, residual carbon, and mixed copper‐oxide phases collectively govern functional performance. By integrating DFT with multimodal bimodal AFM, multivariate PCA, and complementary techniques including SEM, XRD, XPS, nitrogen physisorption (BET), and UV–Vis–NIR spectroscopy, we establish a correlative multiscale mechanistic framework describing this transformation.

### 8.1 DFT

**FIGURE 7:**
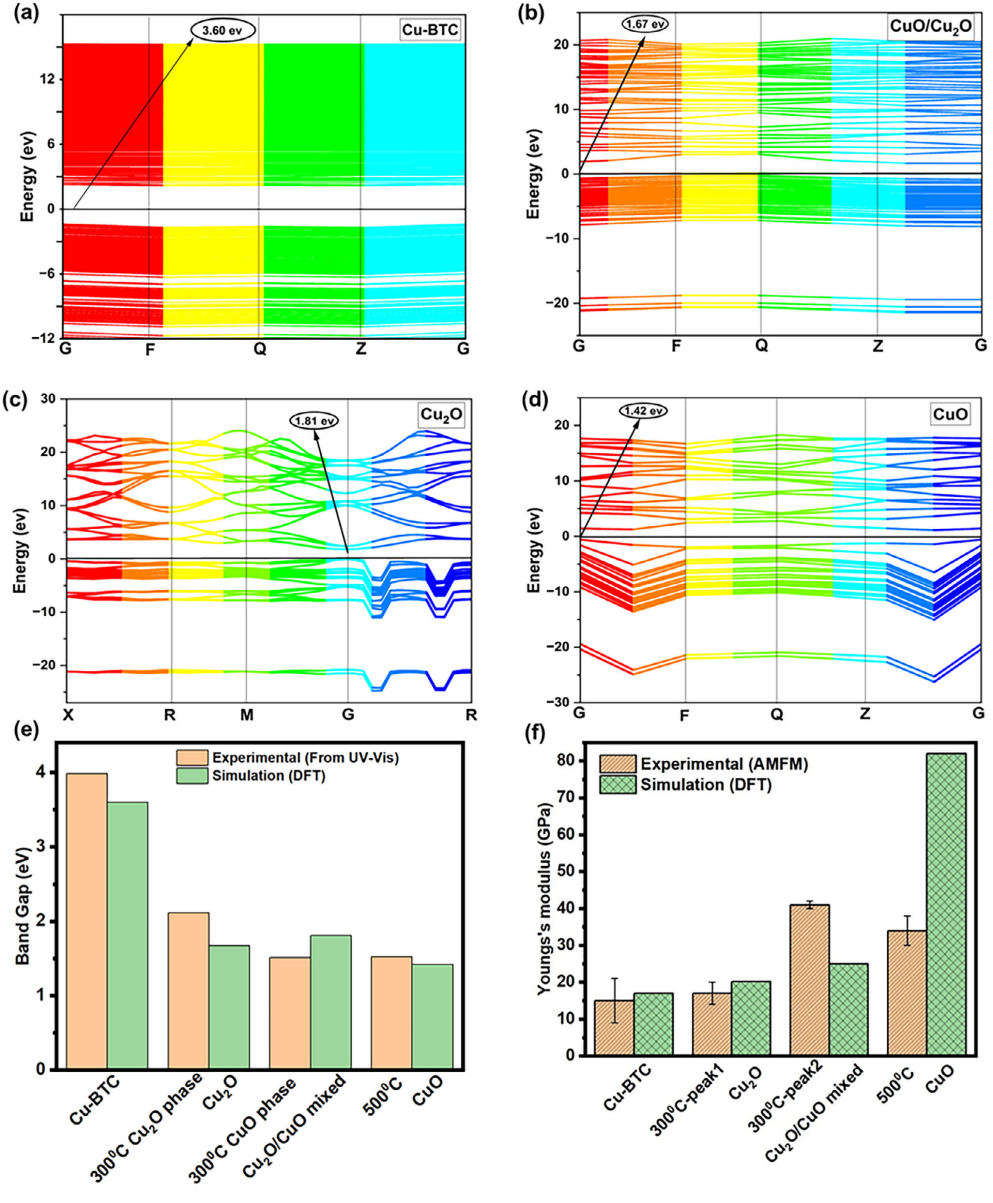
DFT bandgap structure calculations of (a) Cu‐BTC, (b) CuO/Cu_2_O, (c) Cu_2_O, (d) CuO, (e) comparison of our DFT simulation results with our UV‐Vis experimental results, (f) comparison between our DFT simulation results and the AM‐FM AFM experimentally measured Young's modulus. DFT calculations reveal bandgap evolution from 3.60 eV (Cu‐BTC) to lower values in Cu_2_O, CuO, and their heterophase, consistent with experiments, while also showing good agreement between simulated and measured Young's modulus, confirming the electronic and mechanical changes during MOF‐to‐oxide transformation.

DFT calculations were performed using the CASTEP package to investigate the electronic structures of Cu‐BTC, Cu_2_O, CuO, and the intermediate Cu_2_O/CuO heterophase (Figure 7). The resulting hybrid‐DFT bandgaps were 3.60 eV for Cu‐BTC [36], 1.67 eV for the Cu_2_O/CuO heterophase, 1.81 eV for Cu_2_O [37], and 1.42 eV for CuO [38] (Figure 7a–d).

These values closely align with the experimental results obtained from the Tauc plot analysis (Figure S10). The detailed methodology and experimental bandgap plots for Cu‐BTC and its calcined derivatives are provided in Section S4. The analysis confirms the semiconducting behavior of CuO*_x_* and highlights the electronic changes associated with phase evolution and interfacial coupling during the MOF‐to‐oxide transformation. The Young's modulus was also calculated using DFT to assess the theoretical mechanical properties and compared with the experimentally measured values obtained from AM–FM AFM measurements, as presented in Figure 7f.

### 8.2 Stage I: Pristine Cu‐BTC—Soft, Highly Porous, and Strongly Dissipative (Room Temperature)

At ambient conditions, Cu‐BTC consists of Cu(II) paddlewheel nodes bridged by benzene‐1,3,5‐tricarboxylate ligands, forming a highly ordered yet mechanically compliant framework. AM–FM AFM measurements yield a low Young's modulus of 15 ± 6 GPa, sharply distinct from the SiO_2_ substrate (73 ± 3 GPa). AM–AM phase and dissipation maps show large phase shifts (∼16°), indicative of viscoelastic losses and interfacial friction intrinsic to the hybrid organic–inorganic architecture.

PCA analysis reveals strong mechanical uniformity in the system, with Young's modulus, dissipation, and frequency shift forming tightly clustered correlations. In contrast, topography and amplitude show weak correlations, indicating limited coupling between surface morphology and mechanical response. Two distinct dissipation pathways are resolved in AM–AM mode, one associated with surface interactions and the other with bulk viscoelastic relaxation. These findings demonstrate that even the pristine MOF exhibits multiple nanoscale energy dissipation mechanisms.

Nitrogen physisorption (Figure S4) confirms a type‐IV isotherm with a high BET surface area of 1045 m^2^ g^−^^1^ and a narrow pore size distribution centered at 2.6 nm, reflecting a highly uniform microporous network. XRD confirms long‐range crystallinity, XPS verifies Cu(II)–carboxylate coordination, and SEM shows intact faceted crystals, establishing a structurally and chemically pristine starting point.

### 8.3 Stage II: Ligand Decomposition and Mixed Oxide Nanocomposite Formation (∼300°C)

Annealing to 300°C initiates linker decomposition, partial framework collapse, and the nucleation of Cu_2_O with incipient CuO domains. AM–FM stiffness maps resolve three mechanically distinct populations: residual MOF‐rich regions (17 ± 3 GPa), newly formed oxide‐rich domains (41 ± 1 GPa), and the substrate (73 ± 6 GPa), revealing pronounced nanoscale heterogeneity.

Crucially, nitrogen physisorption (Figure S4) shows that although the BET surface area decreases sharply to 33 m^2^ g^−^^1^, the pore size distribution broadens into the mesoporous regime, with a dominant pore diameter of ∼12.1 nm. This mesoporous architecture is templated directly from the parent MOF and forms a well‐connected Cu_2_O‐rich scaffold capable of efficiently redistributing applied stress. This structural motif underpins the anomalously high local modulus observed in AM–FM maps.

XPS analysis provides critical chemical context. While the system becomes increasingly inorganic, a substantial carbon fraction (∼16%–17%) persists, demonstrating that the 300°C material is not a simple oxide but an oxide–carbon nanocomposite. Notably, the highest modulus population coincides with the maximum copper concentration (∼45%), indicating an optimal composite architecture in which a copper‐rich inorganic framework is mechanically reinforced by a residual carbon matrix residing along pore walls and intergranular regions.

AM–AM dissipation maps exhibit contrast inversion between low‐ and high‐frequency modes: MOF‐rich regions dissipate less than the substrate at low frequencies but more at high frequencies, revealing enhanced internal friction associated with partial collapse, oxide nucleation, and interfacial disorder. PCA captures this transition, with increasing coupling between mechanical, dissipative, and morphological observables, reflecting the emergence of mixed‐phase load‐bearing pathways.

XRD shows attenuation of Cu‐BTC reflections and the appearance of broad Cu_2_O/CuO features, while XPS reveals partial reduction from Cu(II) to Cu(I). SEM confirms crystal collapse and surface roughening, identifying this temperature regime as a structurally, chemically, and mechanically transitional state.

### 8.4 Stage III: Crystallization and Densification Into Porous CuO (∼500°C)

At 500°C, conversion to monoclinic porous CuO is completed. AM–FM AFM reveals a homogenized mechanical landscape dominated by a single CuO phase with a modulus of 34 ± 4 GPa. Despite CuO being intrinsically stiffer as a bulk phase, the effective modulus decreases relative to the 300°C intermediate.

This counterintuitive trend is explained by the concurrent evolution of pore architecture and chemistry. BET analysis (Figure S4) shows a further reduction in surface area to 11 m^2^ g^−^^1^, accompanied by significant pore enlargement extending toward the macroporous regime. SEM and AFM reveal particle shrinkage, reduced roughness, and evidence of sintering, all indicative of pore coarsening and partial structural collapse.

XPS confirms exclusive Cu^2^^+^ states but also reveals that residual carbon persists, confirming that even the fully converted material remains an oxide–carbon composite (Figure S3). The loss of fine mesoporosity, combined with transformation‐induced stresses and weakened interparticle contacts, degrades the load‐bearing capacity of the porous network. Consequently, the experimentally measured modulus is substantially lower than the DFT‐predicted value for defect‐free CuO (∼82 GPa [39]).

AM–AM mode 2 dissipation remains elevated, indicating that bulk frictional losses at grain boundaries dominate over surface hysteresis. PCA shows strong clustering and clear variable alignment: stiffness‐related observables form a coherent axis, while dissipation and phase define a distinct damping signature, highlighting the role of polycrystalline texture in governing nanoscale energy loss.

### 8.5 Correlating Nanomechanics With Electronic and Optical Evolution

The nanomechanical evolution is tightly coupled to the electronic transition from an insulator to a semiconductor. UV–Vis–NIR spectroscopy (Figure S8) reveals that pristine Cu‐BTC exhibits weak absorption across the visible range, with light attenuation dominated by scattering rather than true absorption, consistent with its wide bandgap (∼4.0 eV experimentally (Figure S10); ∼3.6 eV from DFT).

At 300°C, Tauc analysis resolves two optical bandgaps (∼2.1 and ∼1.5 eV), corresponding to Cu_2_O and CuO, respectively. This electronic heterogeneity mirrors the mechanically heterogeneous nanocomposite architecture resolved by AFM. Our simplified Cu_2_O/CuO DFT model yields an intermediate bandgap (∼1.67 eV), capturing the electronic signature of heterophase coupling and interface formation.

By 500°C, a single experimental bandgap (∼1.5 eV) emerges, closely matching the DFT value for CuO (∼1.42 eV), confirming that the optoelectronic response is dominated by the oxide phase. The experimental and simulated bandgaps of all samples are presented in Figure 7e. To further validate the optoelectronic properties of the thermally derived phases, the experimental and computational bandgaps were compared with standard literature values (Table 1). As shown, the bandgap of our MOF‐derived porous CuO at 500°C (1.56 eV) aligns closely with the reported literature range for conventionally synthesized CuO (1.44 eV), confirming the successful optoelectronic conversion of the framework. This comparison confirms that the MOF‐templated thermal conversion not only preserves the mesoporous structural hierarchy but also yields functional semiconducting oxides with optical properties highly comparable to standard materials. Electron density distribution maps (Figure S11) show progressive electron delocalization with increasing Cu–O connectivity, driving bandgap narrowing and enhanced light absorption. Absorption and scattering coefficients extracted from fluid‐dispersion measurements confirm that the enhanced optical response arises from genuine absorption rather than scattering artefacts.

First‐principles elastic constants combined with the Voigt–Reuss–Hill model [40] yield moduli of 18 ± 2 GPa (Cu‐BTC), 25 ± 2 GPa (Cu_2_O/CuO), 20 ± 2 GPa (Cu_2_O) [41], and 82 ± 2 GPa (CuO) [39], reproducing the experimental trend obtained from bimodal AFM (Figure 7f). Notably, the experimentally observed high‐modulus population at 300°C (∼41 GPa) exceeds the DFT predictions for ideal phases. It should be noted that our computational models evaluate the idealized Cu‐BTC, Cu_2_O, CuO, and Cu_2_O/CuO heterointerfaces. The residual amorphous carbon network, while experimentally verified via XPS to be present and to provide critical mechanical reinforcement, was not explicitly incorporated into the DFT models due to its inherent structural complexity and disorder. Therefore, the enhanced Young's modulus observed experimentally is interpreted as arising from a synergistic combination of heterophase interactions and the physical reinforcement from the mesoporous carbonaceous scaffold, an effect that lies beyond the scope of idealized crystal simulations.

A comparative overview of the structural, mechanical, and optical evolution across calcination temperatures is provided in Table 2. The transformation from the softer, highly porous Cu‐BTC framework to a mechanically reinforced, optically active porous CuO network underscores the critical role of controlled thermal treatment in tuning multifunctional properties of MOF‐derived oxides.

**TABLE 2:** Summary of key structural, mechanical, and optical parameters of Cu‐BTC and its derivatives P‐CuO*_x_*.

Property	Pristine Cu‐BTC	Calcined at 300^°^C	Calcined at 500^°^C
Phase	Cu‐BTC	Cu_2_O & CuO	CuO
Modulus (GPa)	15 ± 6	17± 2/41 ± 1	34 ± 4
Roughness (nm)	4.5± 2.1	3.3 ± 1.1	1.1 ± 0.3
Optical absorption	Weak	Moderate	Strong
Dissipation (first mode)	High	Low	Medium

## 9 Summary

This mechanistic model illustrates how our integrated multimodal approach resolves the nanoscale complexity of MOF‐to‐porous oxide transformation, capturing the coupled evolution of structure, mechanics, and dissipation across multiple length scales. The phase‐ and mode‐specific responses observed by multifrequency AFM highlight the sensitivity of this technique to heterogeneous nanostructures, providing insights that are inaccessible to conventional, ensemble‐averaged methods.

These results collectively establish a clear structure–property–function relationship in MOF‐derived copper oxides. The effective mechanical response, dissipation behavior, porosity, and bandgap evolution are primarily dictated by nanoscale connectivity, heterophase interfaces, and mesoporous architecture, rather than by bulk phase composition alone. Notably, the 300 °C intermediate represents a mechanically and electronically optimal nanocomposite, wherein copper‐rich oxide scaffolds are reinforced by residual carbon and mesoporosity. In contrast, complete oxidation at 500 °C produces a chemically homogeneous but mechanically softened porous semiconductor due to pore coarsening and transformation‐induced stresses.

This study demonstrates that controlled partial conversion, rather than full calcination, provides a powerful strategy for engineering mechanically robust and optoelectronically active porous oxides. More broadly, it highlights the unique capability of combining multifrequency AFM with hybrid DFT and complementary spectroscopic techniques to resolve transient, functionally relevant states in hybrid materials that are otherwise hidden in phase‐averaged measurements yet are crucial for rational design and optimization of advanced porous oxides.

## 10 Materials and Methods

### 10.1 Material

Copper benzene‐1,3,5‐tricarboxylate (Cu‐BTC), with the chemical formula C_18_H_6_Cu_3_O_12_ and purchased from Sigma Aldrich (catalogue no. 688614), was utilized as the precursor for synthesizing P‐CuO*_x_*. Ethanol served both as the medium for dispersing the material during sample preparation for various characterization methods and as the base fluid for creating dispersions used in optical measurements.

### 10.2 Synthesis

P‐CuO*_x_* was synthesized via the thermal decomposition of Cu‐BTC through a controlled calcination process in a muffle furnace, as described in the literature [15]. Approximately 30 mg of Cu‐BTC powder was uniformly spread in a 60 mm diameter borosilicate Petri dish and subjected to calcination at 300°C and 500°C. The HR was maintained at 5°C/min, and the samples were held at the target temperature for 1 h under ambient air conditions. The resulting powders, P‐CuO*_x_*, were allowed to cool naturally to room temperature and stored in airtight containers for further analysis.

### 10.3 Thermogravimetric Analysis (TGA)

TGA was carried out using a TGA Q500 to evaluate the temperature‐dependent weight loss of the Cu‐BTC during the calcination process. The measurement was performed under an air atmosphere over a temperature range of 25°C–600°C, using a HR of 5°C min^−^^1^.

### 10.4 AFM

Bimodal dual AC AFM was carried out using PPP‐NCH cantilever with first spring constant of 37.87 N/m and the second spring constant of 1178 N/m, and first and second Eigenfrequencies of 300.4 kHz and 1.68 MHz, with free amplitudes of 73 and 1.9 nm, respectively, with a setpoint of 36.5 nm. In the case of bimodal AM‐FM AFM, the free amplitude was set at 146 and 0.5 nm, respectively, with a set point of voltage of 88 nm. To prepare the samples, the powders were first dispersed in ethanol and then spin‐coated onto clean silicon wafer substrates to achieve a uniform and ultrathin particle layer. This technique was employed to minimize particle agglomeration and enable clearer visualization of individual particles. The average surface roughness was calculated from the obtained topography maps using an Asylum Research‐provided software package, Igor‐Pro.

### 10.5 SEM

The surface morphology and microstructural features of the as‐synthesized materials were examined using a HITACHI SU5000 scanning electron microscope (SEM). Imaging was performed under high‐vacuum conditions at an accelerating voltage of 10 kV. For consistency across surface characterization techniques, the same samples prepared for AFM were also utilized for SEM analysis.

### 10.6 XPS

Surface chemical composition and elemental states of the samples were analyzed using XPS on a Thermo Fisher Scientific ESCALAB Xi+ spectrometer. The system was equipped with a monochromatic Al Kα X‐ray source (photon energy = 1486.6 eV, spot size = 650 µm), operated at 15 kV and 15 mA. Survey spectra were collected with a pass energy of 160 eV and a step size of 1 eV, while high‐resolution spectra were obtained at a pass energy of 20 eV and a step size of 0.05 eV. The analyses were performed under ultra‐high vacuum conditions (∼10^−^^9^ mbar). Binding energies were calibrated using the C 1s peak at 284.8 eV, and data analysis was conducted using Thermo Scientific Advantage software. For XPS samples preparation, the powders were dispersed in ethanol, which were then deposited onto silicon wafer substrates using a drop‐casting method, and dried on a hot plate at 75 °C.

The electronic structure of Cu‐BTC and derived P‐CuO*_x_* were analyzed by using ultraviolet photoelectron spectroscopy (UPS) where the energy of the monochrome UV light source was 21.22 eV.

### 10.7 XRD

The crystalline structure and phase composition of the P‐CuO*_x_* powders were examined using a Bruker D8‐Discover X‐ray diffractometer, operated with a Cu Kα radiation source (*λ* = 1.54056 Å) at 40 kV and 40 mA. The diffraction data were collected across a 2θ range of 10°–60°, using a zero‐background sample holder to reduce interference and enhance pattern clarity.

### 10.8 UV–Vis

Ultraviolet–visible–near infrared (UV–Vis–NIR) spectroscopy was employed to investigate the optical properties of Cu‐BTC and its calcined derivatives. The measurements were performed at room temperature using a Lambda 1050+ spectrophotometer, from PerkinElmer Inc, equipped with a 150 mm integrating sphere to capture both transmitted light and any reflected or scattered light, allowing for a more comprehensive analysis of the material's interaction with light. To prepare the samples, fluid dispersions were prepared by dispersing the powdered forms of Cu‐BTC and the calcined samples of 300°C and 500°C into ethanol at a concentration of 0.02 wt%. The dispersions were homogenized using bath sonication for 10 min to ensure uniform particle distribution and minimize agglomeration. The measurements were carried out over the wavelength range of 300–1400 nm using 3 mL of fluid dispersion placed into a four clear windows quartz cuvette with a 10 mm optical path length. For each measurement, 3 mL of the prepared dispersion was transferred into a quartz cuvette with four clear optical windows and a 10 mm path length. Spectral data were recorded over the 200–1500 nm wavelength range.

### 10.9 DFT

Structural relaxations were carried out within the generalized gradient approximation (GGA) using the PBE functional. To obtain accurate electronic bandgaps, single‐point calculations at the Γ point (a high‐symmetry point) were subsequently conducted using hybrid exchange—correlation functionals (B3LYP and HSE06), addressing the well‐known bandgap underestimation inherent to semi‐local GGA approaches [44, 45]. Spin‐polarized antiferromagnetic configurations were explicitly included for CuO, as omission of magnetic ordering led to significant bandgap overestimation [46]. The Cu_2_O/CuO intermediate phase was modelled using a low‐symmetry interfacial configuration constructed from CuO (111) and Cu_2_O (100) crystallographic planes, selected to best reproduce the experimentally observed bandgap characteristics. This heterophase was treated using a layered slab geometry with a vacuum spacing of 6 Å to eliminate spurious interlayer interactions. The B3LYP hybrid functional was employed for Cu‐BTC, Cu_2_O, and CuO, while the mixed Cu_2_O/CuO heterophase was evaluated using the HSE06 functional incorporating 25% Hartree–Fock exchange, reflecting its demonstrated reliability for complex oxide interfaces [47]. All calculations used a plane‐wave cutoff energy of 600 eV, a fine grid scale of 1.5, and an electronic convergence threshold of 1 × 10^−^^6^ eV. Monkhorst–Pack k‐point meshes of 4 × 2 × 2 (Cu_2_O/CuO), 4 × 4 × 4 (Cu_2_O), and 4 × 3 × 1 (CuO) ensured adequate Brillouin‐zone sampling.

## Conflicts of Interest

The authors declare no conflicts of interest.

## Supporting information




**Supporting File**: smll74631‐sup‐0001‐SuppMat.docx.

## Data Availability

The data that support the findings of this study are available on request from the corresponding author.
