# Supplementary material for: Revealing Structure–Property Coupling During Thermal Conversion of Metal–Organic Frameworks
Source: Small. 2026 Jul 22;22(51):e74631. doi: 10.1002/smll.74631 (PMC13568913; doi:10.1002/smll.74631)
Supplement: Supplementary file 1 [file SMLL-22-e74631-s001.docx]

**Revealing structure–property coupling during thermal conversion of metal organic frameworks**

Gourav Bhattacharya^1^, Sameh Khalil^1^, Wajahat Khalid^1^, Indrianita Lionadi^1^, Abhijit

Ganguly^1^, Anna L. Eichhorn,^2^ Christian Dietz^2^, Amir Farokh Payam^1^*, Supriya Chakrabarti^1^*

^1^ Nanotechnology and Integrated Bioengineering Centre, School of Engineering, School of Engineering, Ulster University, Belfast, UK.

^2^ Physics of Surfaces, Institute of Materials Science, Technische Universität Darmstadt, Darmstadt, Germany.

**Supplementary Information**

**Figure S1 (a)** TGA/DTG profiles of Cu-BTC measured under air atmosphere at a heating rate of 5 °C min⁻¹. and (b) calculated percentage retained weight (Blue) and the percentage weight loss of Cu-BTC MOF, Cu-BTC MOF annealed at 300 °C, and Cu-BTC MOF annealed at 500 °C.

**Table S1.** R.M.S Roughness of Cu-BTC MOF and subsequent transformed phases at 300 °C and 500 °C

| **Sample** | **r.m.s roughness (nm)** |
| --- | --- |
| **Cu-BTC MOF** | 4.5±2.1 |
| **Cu-MOF annealed at 300 °C** | 3.3±1.1 |
| **Cu-MOF annealed at 500 °C** | 1.1±0.3 |

**Figure S2** XPS survey spectra of heat-treated Cu-BTC at different temperatures

**Table S2.** Surface elemental composition (%) from XPS analysis for heat-treated Cu-BTC at 1 hour with HR of 5°C min^-1^

| **Sample** | **Elements** | | |
| --- | --- | --- | --- |
|  | **C** | **O** | **Cu** |
| **Cu-BTC** | 51.05 | 39.65 | 9.30 |
| **300 °C** | 16.88 | 37.75 | 45.37 |
| **500 °C** | 15.98 | 41.2 | 42.82 |

**Figure S3** Elemental composition from XPS survey scans. Bar chart showing the atomic percent (at%) of Carbon (C), Oxygen (O), and Copper (Cu) for the pristine Cu-BTC MOF and the samples thermally annealed at 300 °C and 500 °C.

**Table S3.** Peak fitting results of C 1s heat-treated Cu-BTC at 1 hour with HR of 5°C min^-1^

| **Sample** | **Component** | **Binding Energy (eV)** | **FWHM (eV)** | **At.%** |
| --- | --- | --- | --- | --- |
| **Cu-BTC** | C-C | 284.8 | 1.6 | 59.43 |
|  | C-O | 285.96 | 1.67 | 16.54 |
|  | O-C=O | 288.81 | 1.82 | 22.1 |
|  | π- π | 290.93 | 1.42 | 1.93 |
| **300 °C** | C-C | 284.8 | 1.45 | 55.89 |
|  | C-O | 286.11 | 1.5 | 24.32 |
|  | C=O | 288.24 | 2.2 | 19.79 |
| **500 °C** | C-C | 284.8 | 1.55 | 62.31 |
|  | C-O | 286.14 | 1.27 | 23.52 |
|  | C=O | 288.05 | 1.67 | 14.17 |

**Table S4.** Peak fitting results of O 1s heat-treated Cu-BTC at 1 hour with HR of 5°C min^-1^

| **Sample** | **Component** | | **Binding Energy (eV)** | | **FWHM (eV)** | | **At.%** | |
| --- | --- | --- | --- | --- | --- | --- | --- | --- |
| **Cu-BTC** | Cu-O-C | 532.75 | | 2.05 | | 100 | |  |
| **300 °C** | O=Cu | 529.03 | | 1.02 | | 39.06 | |  |
|  | O-H | 530.77 | | 1.71 | | 33.06 | |  |
|  | O-C | 532.62 | | 1.85 | | 27.88 | |  |
| **500 °C** | O=Cu | 529.01 | | 1.04 | | 46.78 | |  |
|  | O-H | 530.78 | | 1.87 | | 32.95 | |  |
|  | O-C | 532.5 | | 1.86 | | 20.27 | |  |

**S1. Porosity and Surface Area Analysis using BET**

Nitrogen physisorption measurements were performed to determine the specific surface area and pore size distribution of the synthesized P-CuOx, following the BET method. The analysis was conducted at liquid nitrogen temperature, and the full isotherm of the adsorption-desorption process was recorded as presented in Figure S4. Before the measurements, samples were degassed under vacuum at 150 °C for 16h to remove any adsorbed contaminants. The obtained full isotherm for all samples can be categorized as type IV based on the international classification of isotherms [1]. Moreover, it is accompanied by a hysteresis loop, that indicates the mesoporous structure for all samples. The BET surface area was calculated from the nitrogen adsorption isotherms in the relative pressure range of p/p_o_ = 0.3, where p_o_ represents the saturation pressure of nitrogen. The Cu-BTC sample presented a high surface area with 1045 m² g⁻¹. However, this value significantly decreased to 33 m² g⁻¹ and 11 m² g⁻¹ for the samples treated at 300 and 500˚C, respectively. Pore size distribution and the total cumulative pore volume, noted in Table S5, were analyzed according to Density Function Theory [2]. This pore size analysis was derived from the amount of gas adsorbed over a range of P/P_0_ from 0.15 to near one, and the total volume of pores was obtained at p/po= 0.95. The pore size distribution of pristine Cu-BTC displayed sharp peaks and exhibited a sharp, narrow pore size distribution with a predominant pore size of 2.6 nm, reflecting uniformity across the sample. Upon calcination at 300 °C, the pore size increased significantly, showing a broader distribution with a most common pore size around 12.1 nm. Further calcination at 500 °C led to an even wider pore size distribution, covering a range within the mesoporous region (<50 nm) and extending into the macroporous range.

**Figure S4** (a-c) N_2_ Adsorption–desorption isotherms of Cu-BTC and P-CuOx at 300 °C and 500 °C, respectively. (d-f) Pore volume and pore width for Cu-BTC and P-CuOx at 300 and 500 °C, respectively.

**Table S5.** Surface area, pore width and total pore volume for samples.

| **Samples** | $\boldsymbol{S}_{\boldsymbol{BET}}\boldsymbol{(}\mathbf{m}^{\mathbf{2}}\mathbf{g}^{\mathbf{-1}}\boldsymbol{)}$ | **Mode Value of Pore width (nm)** | **Pore volume**$\boldsymbol{(}\mathbf{cm}^{\boldsymbol{3}}\mathbf{g}^{\mathbf{-}\mathbf{1}}\boldsymbol{)}$ |
| --- | --- | --- | --- |
| Cu-BTC | 1212 | 2.6 | 0.656 |
| 300 °C | 33 | 12.1 | 0.139 |
| 500 °C | 11 | 22.1 | 0.042 |

**S2. MVA analysis:**

We examined the MVA correlations of the AFM observables (Figures S5–S7), to trace how the nanomechanical response of the Cu-MOF evolves throughout thermal treatment.

In the pristine Cu-MOF, two largely independent interaction regimes are evident. Topography correlates strongly with Virial 2 (0.79) and anticorrelates with Dissipation 1 (–0.81), behaviour that aligns with a hybrid coordination framework where metal nodes dominate the conservative response and the organic linkers accommodate energy dissipation. Meanwhile, the strong clustering of the higher-harmonic parameters which are Amplitude 2, Phase 2, and Dissipation 2—shows that local variations in stiffness and damping arise predominantly from nonlinear viscoelastic processes inherent to the intact, flexible structure.

Upon annealing at 300 °C, the correlation patterns reorganize substantially, consistent with the formation of an intermediate carbonaceous– mixed copper oxide phase. The link between topography and conservative interactions persists (Virial 2, 0.75), yet the fundamental phase becomes much more sensitive to attractive force gradients, as seen in its strong negative correlation with Virial 1 (–0.92). Dissipation 1 aligns almost perfectly with Phase 1 (0.98), indicating a shift in the origin of energy loss. Rather than restricting from the mobility of the organic linkers, dissipation is now dominated by localized relaxation at the emerging mixed copper oxide/carbon interfaces (as most of the linkers had been collapsed due to annealing) and by the onset of structural stiffening within the partially decomposed framework. It is to be mentioned though our mechanical mapping exhibited two distinct regions, the MVA analysis just provided an average correlation.

At 500 °C, the system transitions fully into a carbonaceous CuO network, and the correlation matrix undergoes a striking collapse toward near-unitary values (|r| ≈ 0.93–1.00). This convergence does not imply a loss of porosity; instead, it reflects the development of a highly uniform, nanoporous scaffold. The diverse mechanical signatures characteristic of the MOF phase almost vanishes for this sample. The higher-harmonic observables confirm that the soft, viscoelastic pathways originally provided by organic ligands are no longer present. At this stage, both dissipation and stiffness scale predictably with interaction volume, demonstrating that the mechanical behaviour is governed by the continuous, interconnected network formed during high-temperature annealing.

Overall, this thermal progression reveals a clear trajectory: from the dual soft–hard mechanics of the pristine MOF, through the chemically heterogeneous mixed-oxide intermediate, to the rigid and mechanically uniform porosity of the carbonaceous cupric oxide.

**Figure S5** MVA heat map of Cu-MOF.

 **Figure S6** MVA heat map of Cu-MOF annealed at 300 °C.

**Figure S7** MVA heat map of Cu-MOF annealed at 500 °C.

**UV-Vis-NIR Transmittance and Scattering Study:**

**Figure S8** (a) The transmittance (%T), and (b) The combined transmittance plus scattering (%T+S) of pristine Cu-BTC and the synthesized P-CuO_x_. (c) Absorption coefficients, and (d) Scattering coefficients of pristine Cu-BTC and the synthesized P-CuO_x_.

# **S3. Measurements and calculations of optical properties-**

To evaluate the absorption and scattering coefficients, of the samples, the transmittance and scattering were measured using a UV-VIS-IR spectroscopy; Lambda 1050+ with 150 mm integrating sphere, PerkinElmer Inc in a wavelength range of 300-1500 nm. After loading the sample into a standard cuvette (quartz, 3.5 mL capacity and 1 cm light path length, PerkinElmer Inc), it was placed in the transmission port to measure its transmittance ($T$), and then centrally mounted in the integrating sphere to measure transmittance and scattering combined ($T+S$), see Figure S8a and b. The Figure S9 shows a schematic presentation of T and T+S measurement method using integrating sphere inside the spectrometer.

**Figure S9.** (a) Schematic shows methods of direct measurements of transmittance using the transmission port. (b) Schematic shows methods of direct measurements of transmittance and scattering combined within the integrating sphere, adapted from our previous publication [3].

The attenuation of the incident light, $I_{o}$*,* by absorption and scattering while travelling through the sample can be expressed mathematically as follows:[3]

$$I\left( x \right)=I_{o}e^{-\mu_{e}x}$$

(1)

- $I\left( x \right)$: light at any point *x*, which is equivalent to the transmitted light
- $\mu_{e}$: extinction coefficient
- $x$: the path length that the light has travelled through

Since the transmittance is the ratio between the transmitted light to the incident light, the extinction coefficient can be also determined:

$$T=\frac{I\left( x \right)}{I_{o}}=e^{-\mu_{e}x}$$

(2)

$$\mu_{e}=-\frac{lnT}{x}$$

(3)

The light absorbed ($I_{a}$) at a given wavelength within a fluid depth (*x*) within the sample can be calculated by the integral over the total path length from $x=0$ to $x=L$ multiplied by the absorption coefficient ($\mu_{a}$):

$$I_{a}\left( L \right)=\int_{0}^{L} \mu_{a}I\left( x \right)\cdot dx$$

(4)

Substituting by Equation S1:

$$I_{a}=\int_{0}^{L} {\mu_{a}I_{o}e}^{-\mu_{e}x}\cdot dx$$

(5)

$$I_{a}=\mu_{a}I_{o}\int_{0}^{L} e^{-\mu_{e}x}\cdot dx$$

(6)

$$I_{a}=\mu_{a}I_{o}\left( \frac{1-e^{-\mu_{e}L}}{\mu_{e}} \right)$$

(7)

In the case of liquid samples as in this work, light is either absorbed ($I_{a}$), scattered ($I_{s}$) or transmitted ($I_{t}$) and therefore:

$Abs+S+T=1$

(8)

- $Abs$: fraction of light absorbed (absorptance) $=I_{a}/I_{o}=1-\left( S+T \right)$, and,
- $S$: fraction of light scattered $=I_{s}/I_{o}$.

The absorption coefficient can be then determined by rearranging Equation 7 and substituting by the measured value of ($T+S$) as follows:

$$\mu_{a}=\left( \frac{\mu_{e}}{1-e^{-\mu_{e}L}} \right)\frac{I_{a}}{I_{o}}$$

(9)

$$\mu_{a}=\left( \frac{\mu_{e}}{1-e^{-\mu_{e}L}} \right)\left( 1-\left( S+T \right) \right)$$

(10)

and then we can substitute by the extinction coefficient in Equation 3 into Equation 10 to reach the final formula of the absorption coefficient as a function of the measured optical properties as follows:

$\mu_{a}=\left( 1-\left( S+T \right) \right)\left( \frac{\left( -\frac{lnT}{L} \right)}{\left( 1-e^{-\left( -\frac{lnT}{L} \right)L} \right)} \right)$

(11)

$$\mu_{a}=\left( 1-\left( S+T \right) \right)\left( \frac{-lnT}{L\left( 1-T \right)} \right)$$

(12)

$$\mu_{a}=-\frac{1}{L}\left( \frac{1-\left( S+T \right)}{1-T} \right)lnT$$

(13)

Consequently, the scattering coefficient, $\mu_{s}$, can be simply determined by subtracting the absorption coefficient from the extinction coefficient.

$$\mu_{s}= \mu_{e}-\mu_{a}$$

(14)

**S4. Band gap determination using Tauc Plot:**

From the absorption coefficient, Tauc plot could be generated for band gap calculation. For semiconductor, band gap could be determined according to relation ${(\alpha h\nu)}^{n}=k (h\nu- E_{g})$ [4], where α is the absorption coefficient, h is Planck’s constant, $\nu$ is the frequency of the absorbed light, K is a constant, and E_g_ is the optical band gap energy. The value of n could be 2 or ½ for direct and indirect electron transition respectively. The plot was created by plotting a function of the absorption coefficient as a function of photon energy. The graph generated by plotting ${(\alpha h\nu)}^{2}$ against $(h\nu$) for a direct band gap, and the value of E_g_ could be determined by obtaining the tangent to the graph and identify the point where it intercepts the abscissa, as shown in Figure S10.

**Figure S10** Obtained band gaps from the Tauc plot for (a) Cu- BTC MOF, (b) Cu-BTC MOF annealed at 300 ^0^C, and (c) Cu-BTC MOF annealed at 500 ^0^C.

**DFT simulated Electron density distribution maps:**

**Figure S11** DFT Electron Density Difference calculation (a,b) Cu-BTC (c,d) CuO/Cu₂O (e,f) Cu_2_O and (g,h) CuO.

**S5. Determination of work function and construction of energy band diagram**

Ultraviolet photoelectron spectroscopy (UPS) was employed to determine the work function, valence band maximum, and conduction band minimum of Cu-BTC and the Cu-BTC-derived P-CuO_x_ samples calcined at 300 and 500 °C. The band gap energies were estimated from Tauc plots (Figure S10), as detailed in Section S4 of the Supporting Information. These experimentally derived energy parameters were subsequently used to construct the energy band diagrams of all samples. To gain insight into the depth-dependent electronic properties, UPS measurements of the 300 °C sample were carried out before and after sputtering, which enabled the distinction between the surface and subsurface electronic structures. The UPS spectra were used to extract the cut-off energy (E_Cut-off_), and the valence band on-set energy (E_On-set_), as shown in Figure S12. In the UPS spectrum, the onset energy, commonly referred to as the valence band edge, corresponds to the lowest binding energy at which photoemitted electrons are detected from the sample surface. This feature represents the maximum energy level of electrons within the valence band that can overcome the surface potential barrier and escape into the vacuum for detection. Conversely, the high binding energy cut-off of the UPS spectrum defines the maximum kinetic energy range of detectable photoelectrons. This cut-off is governed by the energy of the incident ultraviolet photons (21.22 eV) and the work function (φ) of the material. The work function of a material is the energy difference between its Fermi energy (E_F_) and the vacuum energy level (E_Vac_) and could be calculated as; *φ* = *hν* −(E_Cut-off_ *−* E_F_) [5], where *hν* is energy of the incident photon which is 21.22 eV. The valence band maximum (E_V_) was subsequently estimated from the work function and the on-set energy, while the conduction band minimum (E_C_) was calculated by combining the valence band position with the optical band gap obtained from the Tauc plots (Figure S10). Analysis of the UPS spectra (Figure S12) revealed onset and cut-off energies of 1.90 and 18.60 eV, respectively for Cu-BTC. For the Cu-BTC-derived P-CuO_x_ sample calcined at 300 °C, the onset & cut-off energies were 0.60 & 17.45 eV before sputtering and 0.42 & 17.98 eV after sputtering, indicating changes in the near-surface electronic structure. The Cu-BTC-derived P-CuO_x_ sample calcined at 500 °C exhibited onset and cut-off energies of 0.57 and 17.53 eV, respectively. From these measurements, work functions of 2.62 eV (Cu-BTC), 3.77 eV (300 °C, before sputtering), 3.24 eV (300 °C, after sputtering), and 3.69 eV (500 °C) were calculated which indicate the influence of thermal treatment on the electronic properties of the materials. Using these experimentally derived energy parameters, energy-level band diagrams were constructed for Cu-BTC, the 300 °C sample before and after sputtering, and the 500 °C P-CuO sample, as shown in Figure S13. The 300 °C sample was therefore represented by two surface-sensitive diagrams corresponding to the CuO-enriched outer surface before sputtering and the Cu₂O-rich subsurface/core revealed after sputtering.

**Figure S12**. UPS spectra of (a) Cu-BTC, (b&c) Cu-BTC-derived P-CuOx calcined at 300 °C before and after sputtering respectively and (d) Cu-BTC-derived P-CuO calcined at 500 °C.

**Figure S13.** Energy-level band diagrams of (a) Cu-BTC, (b&c) Cu-BTC-derived P-CuOx calcined at 300 °C before and after sputtering, respectively, and (d) Cu-BTC-derived P-CuO calcined at 500 °C.

**Reference**

1. Sing, K. S. W. *et al.* Reporting Physisorption Data for Gas/Solid Systems. in *Handbook of Heterogeneous Catalysis* (eds Ertl, G., Knözinger, H., Schüth, F. & Weitkamp, J.) 1217–1230 (Wiley, 2008). doi:10.1002/9783527610044.hetcat0065.

2. Seaton, N. A. et al. A new analysis method for the determination of the pore size distribution of porous carbons from nitrogen adsorption measurements. *Carbon* **27**, 853–861 (1989).

3. Moghaieb, H. S. et al. Efficient solar-thermal energy conversion with surfactant-free Cu-oxide nanofluids. Nano Energy 108, 108112 (2023).

4. Tauc, J. *Amorphous and Liquid Semiconductors*, Springer International Publishing, London, UK 1974.

5. Kim, J. W. et a. Work function measurement by ultraviolet photoelectron spectroscopy: Versailles project on advanced materials and standards interlaboratory study. *J. Vac. Sci. Technol. A* 41, 053211 (2023).
